# Gyroscope Denoising Algorithm Based on EMD-SSA-VMD Double-Layer Decomposition

**DOI:** 10.3390/s26041367

**Published:** 2026-02-21

**Authors:** Chuanqian Lv, Yaohong Zhao, Fangzhou Li, Haibo Luo

**Affiliations:** 1 Key Laboratory of Opto-Electronic Information Processing, Chinese Academy of Sciences, Shenyang 110016, China; lvchuanqian@sia.cn (C.L.); zhaoyaohong@sia.cn (Y.Z.); lifangzhou@sia.cn (F.L.); 2 Shenyang Institute of Automation, Chinese Academy of Sciences, Shenyang 110016, China; 3 University of Chinese Academy of Sciences, Beijing 100049, China

**Keywords:** MEMS gyroscope, empirical mode decomposition, sparrow search algorithm, variational mode decomposition, power spectrum entropy

## Abstract

To reduce random errors effectively and improve measurement precision in MEMS gyroscopes, we establish a dual-layer noise suppression method named EMD-SSA-VMD. The algorithm is grounded in empirical mode decomposition (EMD) and variational mode decomposition (VMD), incorporating the sparrow search algorithm (SSA) and entropy theory. The process starts by breaking down the signal into a series of intrinsic mode functions (IMFs) and a residual via EMD. By calculating the power spectral entropy (PSE) of IMFs, we can sort the signal components into three categories: noise signals, mixed signals, and effective signals. The mixed signals then undergo VMD processing, where SSA optimizes the key decomposition parameters. The sample entropy (SE) of the IMFs from VMD is computed to distinguish between actual signal components and noise. Finally, we combine all valuable signals to reconstruct the denoising signal. MATLAB(R2024b) simulation results show that this algorithm improves both the Signal-to-Noise Ratio (SNR) and the Root Mean Square Error (RMSE), demonstrating a more efficient removal of noise. Experiments on actual gyroscope data confirm these improvements, yielding higher SNR and a waveform that closely matches the original signal. This proves the algorithm’s practical value in engineering applications.

## 1. Introduction

In recent years, micro-electro-mechanical systems (MEMS) have developed rapidly owing to their compact size, high cost-effectiveness, low power consumption, large-scale production, and easy integration. As a result, these sensors have become core devices in inertial navigation systems. They are widely used in fields like navigation, aerospace, the military, and autonomous driving [1,2]. However, the inherent physical structure and manufacturing precision of MEMS sensors lead to large output errors. The main sources are random errors and deterministic errors. We can compensate deterministic errors, such as constant bias, mounting misalignment, and alignment issues, through calibration. In contrast, random errors include bias instability, random drift, and high-frequency noise. These are easily affected by the structure, operational environment, and system circuitry. The strong nonlinear and random nature of MEMS sensors limits their accuracy. Therefore, using denoising algorithms to reduce random errors is crucial for improving the measurement precision of MEMS gyroscopes [3,4].

Researchers have proposed various methods to address random errors in MEMS gyroscopes. The most commonly used approaches can be broadly categorized into modeling compensation, wavelet threshold denoising (WTD), and modal decomposition.

Traditional denoising methods eliminate noise by employing digital filters with different frequency bands. Zhang [5] utilized a low-pass filter to reduce measurement noise in MEMS sensors. Li [6] designed a finite impulse response low-pass digital filter for the measurement and denoising of gyroscopes. These methods are based on classical filtering theory, which relies on the premise that the signal and noise spectra do not overlap. Parameters such as the filter cutoff frequency, passband, and stopband are typically determined on the basis of requirements or empirical knowledge. Consequently, when the signal and noise spectra severely overlap, the denoising effectiveness is often compromised. Modeling compensation methods use prior knowledge of signals and noise to establish stochastic error models, which are then filtered and compensated through techniques such as Wiener filtering or Kalman filtering [3]. However, the efficacy of these approaches is highly sensitive to the precision of the established error model. Random errors are characterized by their non-stationarity, weak linearity, and slow time-varying behavior, which may lead to differences between the error model and the actual system, ultimately impairing the effectiveness of filtering and compensation [7].

WTD suppresses noisy signals by using appropriate wavelet bases and setting appropriate threshold and decomposition scales. The multiscale analysis enabled by the wavelet transform allows for the effective separation of signals across distinct frequency bands [8]. Zhang [9] proposed a MEMS gyroscope denoising method that combined WTD with an improved Elman neural network to eliminate random errors. Hu [10] introduced a technique that used a wavelet transform to remove high-frequency noise. Subsequently, he applied a time series model integrated with a Sage–Husa adaptive Kalman filter to eliminate low-frequency components. However, the dependence of denoising outcomes on the choice of decomposition parameters introduces substantial difficulty in signal processing applications [11,12]. In complex real-world scenarios, WTD lacks applicability.

Modal decomposition provides an adaptive approach for handling nonlinear and nonstationary signals. It does not require establishing an error model or selecting decomposition parameters on the basis of signal characteristics. The commonly used methods include EMD and VMD [13,14,15,16]. Zhang [17] used EMD to reveal the intrinsic frequencies of fiber optic sensors, thereby enhancing the sensors’ anti-interference capability and data accuracy. Guo [18] combined adaptive sliding averaging with EMD to achieve rapid signal denoising for MEMS gyroscopes. However, EMD is susceptible to issues such as modal aliasing [19]. To address modal aliasing, researchers have proposed several improved methods [20,21,22,23,24], such as EEMD and CEEMD. Nevertheless, these methods cannot entirely eliminate the white noise and aliasing phenomena generated during decomposition, failing to resolve the inherent limitations of EMD. Building upon EMD, K. Dragomiretskiy proposed a quasi-orthogonal and adaptive signal processing method known as VMD. VMD decomposes signals into noniterative variational modes with strong robustness [25]. VMD features a more rigorous mathematical model, effectively suppressing modal aliasing [26]. Xu and Lei [27] applied VMD to ground-penetrating radar (GPR) signals with nonstationary characteristics and reported that VMD could effectively remove noise from GPR signals embedded in strong noise backgrounds, effectively overcoming the influences of modal aliasing and endpoint effects. Cao [28] implemented VMD for denoising MEMS high-G shock accelerometers, achieving favorable noise reduction outcomes. However, VMD requires presetting the number of decomposition modes *K* and the penalty parameter α on the basis of experience. These two parameters directly impact the VMD processing results. However, the effectiveness of VMD is highly dependent on the manual configuration of the mode number *K* and penalty parameter α, both of which critically influence the outcome and speed. Consequently, the selection of VMD parameters is often combined with optimization algorithms. To address the parameter selection in VMD, the slime mold algorithm (SMA) was employed in [29] for its optimization, followed by wavelet denoising of the decomposed IMFs, achieving effective noise identification and elimination. The authors in [30,31,32,33] also applied optimization algorithms to the adaptive search of VMD parameters. The denoising performance of the VMD algorithm is effectively improved after parameter optimization. Therefore, current research focuses primarily on suppressing modal aliasing in EMD and optimizing the parameters of VMD.

To address the above issues, we introduce a gyroscope signal denoising algorithm that integrates EMD, VMD, and the sparrow search algorithm (SSA). This approach first employs EMD to decompose the original signal into its constituent IMF components and then calculates the power spectrum entropy (PSE) of each IMF. On the basis of their PSE values, the IMFs are categorized into noise signals, mixed signals containing both noise and useful information, and useful signals. The mixed components are further decomposed via VMD, where the SSA optimizes its key parameters (*K* and *α*), with envelope entropy (EE) serving as the objective function. The sample entropy (SE) of the resulting IMFs from VMD is subsequently computed to screen and eliminate the noise components. Finally, all useful IMFs obtained from both the EMD and VMD stages are used for signal reconstruction. The proposed method focuses on maximizing denoising accuracy. Due to the iterative nature of the optimization algorithm, the computational cost is relatively high. Therefore, the primary scope of this method is offline post-processing, such as high-precision mapping and post-mission trajectory analysis, rather than real-time positioning. This paper presents the main contributions as follows:

1. A dual-layer decomposition architecture combining EMD and VMD. Adding VMD after EMD compensates for the drawbacks of EMD modal aliasing. By applying VMD only to the mixed signals after EMD, the optimization target for VMD is reduced. This combined EMD-VMD structure significantly enhances both the accuracy and speed of the overall signal processing procedure.
2. A dual screening mechanism for IMFs based on entropy theory. The first screening uses PSE to classify the IMFs obtained from EMD, whereas the second screening employs SE to evaluate the IMFs obtained from VMD. This dual screening mechanism effectively separates noise components from the IMFs.
3. Parameter optimization for VMD based on the SSA. The VMD parameters are determined by the SSA, with the objective of minimizing the EE. This method not only eliminates the reliance on manual parameter tuning but also refines the overall denoising process, significantly enhancing the algorithm’s practical applicability.

The organization of this paper is summarized below: Section 2 offers a description of related methods for gyroscope denoising. Section 3 details the design of the EMD-SSA-VMD algorithm. Section 4 presents and analyzes the results of the experiment. Finally, Section 5 presents the concluding remarks.

## 2. Related Works

### 2.1. Gyroscope Signal Output Model

The errors of the MEMS gyroscope mainly include scale factor errors, axis deviations, gyroscope zero drift, and measurement errors. According to the basic characteristics of the MEMS gyroscope, its measurement model can be expressed as follows [34]:

(1) $$\omega_{meas}(t)=\left(A+B\right)\cdot \omega_{real}(t)+\omega_{real}(t)+b_{0}+b(t)+\varepsilon .$$

Here, $\omega_{meas}(t)$ is the measurement signal and $\omega_{real}(t)$ is the true signal. A is the scaling factor error coefficient, B is the nonorthogonal error coefficient caused by axis deviation, and $b_{0}$ is the constant zero bias error. These are deterministic errors that can be compensated through calibration. b(t) is the random walk error, and ε is the random noise during measurement. This type of error is random and is the main factor limiting the performance of MEMS gyroscopes. The calibrated model can be expressed as

(2) $$\omega_{meas}(t)=\omega_{real}(t)+b(t)+\varepsilon .$$

Physically, $\omega_{meas}(t)$ represents the measured angular velocity (typically in units of °/s or rad/s). This signal is composed of the true angular velocity and the noise. It is important to note that while the internal sensing element generates a capacitive signal proportional to the Coriolis force, the output $\omega_{meas}(t)$ processed here is the digital sequence converted from that physical quantity. In modeling compensation methods, b(t) is typically modeled by a first-order Gaussian–Markov process, and ε is commonly represented by Gaussian white noise. Javad Abbas [34] proposed a method that applied an autoregressive model of long-term errors in MEMS gyroscopes to Kalman filtering, which reduced the output error of gyroscope signals. Furthermore, Li [35] achieved noise suppression by integrating a Kalman filter with an adaptive sliding window filter. Although these methods reduce the dependence on the error model, the precision of the model itself continues to be critical. In complex practical application scenarios, accurately constructing a mathematical model for noisy gyroscope signals poses significant challenges.

### 2.2. Empirical Mode Decomposition

EMD overcomes the limitations of modeling compensation and WTD by employing a fully data-driven decomposition process. Traditional EMD uses iterations of extreme points and envelope lines to decompose signals, as shown in Algorithm 1.
**Algorithm 1.** EMD steps.1. Initialization parameters: $R_{0}=X(t),i=1$2. Repeat: (a). Initialization: $h_{0}=R_{i-1}(t),j=1;$ (b). Search for all maximum and minimum values of $h_{j-1}(t)$ and form their respective envelope lines: U, L. (c). Calculate the mean of U and L: $m_{j-1}(t)=\left(U+L\right)/2$; (d). $h_{j}(t)=h_{j-1}(t)-m_{j-1}(t)$; (e). If $h_{j}(t)$ satisfies the IMF condition, ${\mathrm{IMF}}_{i}(t)=h_{j}(t)$. Otherwise, j=j+1, return (b). 3. $R_{i}(t)=R_{i-1}(t)-{\mathrm{IMF}}_{i}(t)$. If $R_{i}(t)$ satisfies the residual condition, the decomposition is over, and $R_{i}(t)$ is the residual. Otherwise, i=i+1, return step 2.

The IMF represents a scale of the original signal, revealing its physical nature. It is not a simple superposition but rather a generalized harmonic with a time-varying amplitude and frequency. The original signal can be perfectly reconstructed without loss by summing all the IMF components along with the residual. Consequently, the signal after EMD can be expressed as

(3) $$X\left(t\right)=\sum_{i=1}^{I}{IMF}_{i}(t)+R(t).$$

Here, ${IMF}_{i}(t)$ is the *i*-th IMF, reflecting the local characteristics of different frequencies in the original signal; I is the number of IMFs; and $R\left(t\right)$ is the residual after decomposition, representing both the signal’s trend error and its low-frequency error.

### 2.3. Variational Mode Decomposition

VMD solves decomposition problems through a variational process that incorporates Wiener filtering, the Hilbert transform, and frequency mixing theory. By introducing a mathematical optimization model with a regularization term, it suppresses modal aliasing and enhances decomposition accuracy and robustness [36]. The signal is separated into a collection of IMFs, each possessing a specific frequency and a limited bandwidth.

VMD separates the signal S(t) into a collection of *K* IMFs. On the basis of the modulation criterion, the IMF is redefined as a harmonic signal u(t), as shown in Equation (4):

(4) $$u_{k}(t)=A_{k}\left(t\right)cos\left(\varphi_{k}\left(t\right)\right).$$

Here, $u_{k}(t)$ is the *k*-th IMF, $A_{k}\left(t\right)$ is the amplitude of $u_{k}(t)$, and $\varphi_{k}\left(t\right)$ is the phase function of $u_{k}(t)$. $u_{k}(t)$ modulates its spectrum to the specific baseband $\omega_{k}$ via the Hilbert transform. The bandwidth is limited by the $L^{2}$ norm of the time derivative and its gradient. With the constraint that the sum of all $u_{k}(t)$ equals the original signal S(t), the model is expressed as follows:

(5) $$\left\{\begin{array}{l} \underset{\left\{u_{k}\},\{\omega_{k}\right\}}{min}\left\{\sum_{k=1}^{K}{∥\partial_{t}\left[\left(\delta \left(t\right)+\frac{j}{\pi t}\right)*u_{k}\left(t\right)\right]e^{-j\omega_{k}t}∥}_{2}^{2}\right\}\\ s.t\;\sum_{k=1}^{K}u_{k}=S \end{array}\right..$$

Here, $\partial_{t}$ is the gradient; $\omega_{k}$ is the center frequency of each IMF; and $\delta \left(t\right)$ is a Dirichlet function.

The constrained problem is difficult to solve directly. Thus, a punishment parameter α and an augmented Lagrange operator λ(t) with strong constraint-handling capability are introduced to convert it into an unconstrained formulation, as shown in Equation (6).

(6) $$L\left[\{u_{k}\left(t\right)\},\{\omega_{k}\},\lambda \left(t\right)\right]=\alpha \sum_{k=1}^{K}{∥\partial_{t}\left[\left(\delta \left(t\right)+j/\pi t\right)\times u_{k}\left(t\right)\right]e^{-j\omega_{k}t}∥}_{2}^{2}+\;{∥S\left(t\right)-\sum_{k=1}^{K}u_{k}\left(t\right)∥}_{2}^{2}+\left\langle \lambda \left(t\right),S\left(t\right)-\sum_{k=1}^{K}u_{k}\left(t\right)\right\rangle .$$

Here, α regulates the modal bandwidth and ensures constraint fulfillment. The VMD operates as described in Algorithm 2.
**Algorithm 2**. VMD steps.Initialization parameters: n = 0, $\left\{u_{k}^{1}\right\},\;\left\{\omega_{k}^{1}\right\},\;\left\{\lambda^{1}\right\}$ = 0;Iterative process:   n = n+1   For k = 1:K   Update $u_{k}$ in the inner loop:
(7)$${\hat{u}}_{k}^{n+1}\left(\omega \right)=\frac{\hat{s}\left(\omega \right)-\sum_{i\neq k}{\hat{u}}_{i}\left(\omega \right)+\hat{\lambda }\left(\omega \right)/2}{1+2\alpha {\left(\omega -\omega_{k}\right)}^{2}}$$   Update
(8)$$\omega_{k}:\omega_{k}^{n+1}=\frac{\int_{0}^{\infty }\omega {\left|{\hat{u}}_{k}\left(\omega \right)\right|}^{2}}{\int_{0}^{\infty }{\left|{\hat{u}}_{k}\left(\omega \right)\right|}^{2}}$$   End   Update $\lambda \left(t\right)$ by dual ascent:    
(9)${\hat{\lambda }}^{n+1}\left(\omega \right)={\hat{\lambda }}^{n}\left(\omega \right)+\tau \left(\hat{s}\left(\omega \right)-\sum_{k}{\hat{u}}_{k}^{n+1}\left(\omega \right)\right)$Until the condition is met:
(10)$$\sum_{k}{\left\|u_{k}^{n+1}-u_{k}^{n}\right\|}_{2}^{2}/{\left\|u_{k}^{n}\right\|}_{2}^{2}<\varepsilon$$

The VMD process requires optimization of all the modes. Several scholars have employed optimization algorithms, including the grasshopper optimization algorithm [30], the rime optimization algorithm [31], the gray wolf optimizer [35], and multi-objective particle swarm optimization [33], to optimize VMD parameters. The denoising performance of VMD significantly improves after parameter optimization, but these algorithms have greater complexity and are not suitable for real-time decomposition.

**Figure 1:**
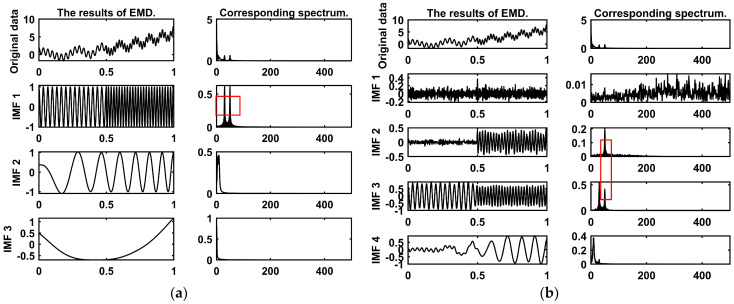
The manifestation of modal aliasing: (**a**) The first type: modes with different frequencies appearing in the same IMF. (**b**) The second type: modes with similar frequencies appearing in different IMFs.

EMD and VMD provide denoising ideas through signal decomposition, but there are still some problems. EMD rapidly decomposes signals through an iterative sifting process, which can easily lead to modal aliasing. Modal aliasing manifests in two forms, as shown in Figure 1: modes with different frequencies appearing in the same IMF, and modes with similar frequencies appearing in different IMFs. This phenomenon causes the IMF to lose its unique scale characteristics, directly undermining the accuracy of signal analysis. Although VMD suppresses modal aliasing by introducing regularization terms, it has shortcomings in real-time decomposition. Therefore, this paper proposes a dual-layer decomposition algorithm named EMD-SSA-VMD. It has a dual-layer decomposition and screening mechanism, which can efficiently and accurately denoise gyroscope signals.

## 3. Methodology

In this section, we introduce the details of the EMD-SSA-VMD algorithm. The algorithm consists of four main components: EMD, IMF screening based on PSE, signal decomposition based on SSA-VMD, and IMF screening based on SE. Traditional modal decomposition approaches typically assume that noise is primarily concentrated in high-frequency IMFs and thus removes it directly. However, as analyzed in Section 2.2, EMD suffers from significant modal aliasing. Consequently, certain IMFs may simultaneously contain both noise and useful components. The mixed IMFs are complicated to handle. If they are retained, the noise will also be retained; if they are discarded directly, some valid information will be lost. In contrast, as discussed in Section 2.3, VMD effectively separates modes of different frequencies by limiting the bandwidth, thereby suppressing modal aliasing. To leverage these complementary strengths, we design a dual-layer structure using EMD in conjunction with VMD. The SSA is employed for optimization. Furthermore, PSE and SE are introduced as criteria for IMF screening at their respective layers. The raw MEMS gyroscope signal contains wide-band noise and complex trends. Directly applying SSA-VMD to the raw signal faces two challenges. First, the optimization search space is too large. The SSA may fall into local optima. Second, the computational cost is excessive. We use EMD as a pre-filter. EMD is parameter-free and fast. It decomposes the raw signal. We use entropy to identify the mixed component. We apply the optimized SSA-VMD only to this mixed component. In this framework, EMD simplifies the target signal for VMD. VMD then focuses on resolving the specific mode-mixing problem within the mixed component. This combination improves both efficiency and decomposition accuracy.

### 3.1. IMF Screening Based on PSE

As analyzed in Section 2.2, PSE can be used to screen the IMFs obtained from EMD. PSE obtains the energy distribution of signals from the frequency domain without setting parameters, demonstrating strong representational capability. Compared with other types of information entropy, PSE requires fewer computations and can better reflect the distribution of signal energy. Furthermore, the PSE is directly related to the SNR and the spectral complexity of the signal. High-noise power signals have a relatively scattered spectral distribution, with the PSE approaching 1. Pure component signals have a spectral distribution concentrated at a few frequency points, with the PSE approaching 0. The normalized PSE of a signal with length *N* is expressed as

(11) $$PSE=-\sum_{i=1}^{N}P_{i}\cdot ln{(P}_{i}+\delta )/lnN.$$

Here, $P_{i}$ is the normalized power spectral and δ is a small positive constant. This study adaptively determines thresholds according to the PSE’s statistical characteristics. Specifically, the noise threshold is derived by adding one standard deviation to the mean, whereas the useful signal threshold is obtained by subtracting it. There are three types of IMFs: noise IMFs, mixed IMFs, and useful IMFs. This classification enables targeted processing for each type. We directly eliminate the noise IMFs. For the mixed IMFs, we perform further VMD to separate noise from useful components. The useful IMFs are retained for subsequent signal reconstruction. This IMF screening mechanism allows for accurate discrimination between noise and useful signaling components.

### 3.2. Mixed Signal Decomposition Based on the SSA-VMD

When performing VMD on mixed signals, this study employs the SSA to automatically determine *K* and *α*. The SSA is adopted as an efficient swarm intelligence technique developed by Xue et al. [37] and benefits from fewer optimization parameters, rapid convergence, low complexity, and ease of real-time implementation. The SSA classifies the sparrow population with the aid of a fitness function to achieve a synergy of global and local optimization. In this work, EE is adopted as the fitness function. We set the minimization of EE as the optimization objective. EE is a measurement method for evaluating signal complexity and uncertainty. It is particularly suitable for handling nonstationary signals. Compared with other information entropies, the EE and SSA have a higher degree of compatibility, along with enhanced noise resistance and robustness. Moreover, EE requires no prior knowledge and offers higher computational efficiency.

The IMF components $u_{k}\left(t\right)$ obtained from the VMD of the signal are subjected to the Hilbert transform to derive their analytic signals $U_{k}\left(t\right)$. The envelope function and EE are then extracted and normalized as follows:

(12) $$U_{k}\left(t\right)=u_{k}\left(t\right)+i\cdot H\left\{u_{k}\left(t\right)\right\}.$$

(13) $$a_{k}(t)=\sqrt{{u_{k}}^{2}(t)+H{\left\{u_{k}(t)\right\}}^{2}}.$$

(14) $$P_{k}(t)=a_{k}(t)/\sum_{t=1}^{N}a_{k}(t).$$

(15) $$E_{k}\;=-\sum_{t=1}^{N}P_{k}(t)\cdot \ln(P_{k}(t)).$$

Here, $a_{k}\left(t\right)$ is the amplitude envelope, $P_{k}\left(t\right)$ is the normalized expression, and $E_{k}$ is the EE of the *k*-th IMF. The large *K* leads to over-decomposition, resulting in the generation of spurious modes. Conversely, the small *K* causes under-decomposition, failing to effectively separate essential signal information. A large *α* restricts the bandwidth of the modes too tightly, potentially causing the loss of signal details, whereas a small *α* results in an excessively wide bandwidth, which may introduce noise or artifacts. Therefore, we define the search ranges for these parameters prior to the optimization process. *K* ∈ [3, 12], *α* ∈ [100, 3000]. The optimization steps for VMD parameters are shown in Algorithm 3.
**Algorithm 3.** VMD parameter optimization algorithm steps.Initialization parameters: *K*: Number of decomposition mode; *α*: The penalty parameter *IC*: Iteration count; *PN*: Number of discoverers; *SN*: Number of Scouts; $R_{2}$: Early-warning value; *N*: Number of sparrows1: The VMD is performed according to *K* and α. 2: While (t < IC)     Rank the fitness to identify the optimal and poorest individuals.     $R_{2}$ = rand (1)     For i = 1: PN         Adjust each sparrow’s position by Equation (16).
(16)$$x_{i,j}^{t+1}=\left\{\begin{array}{c} x_{i,j}^{t}\cdot exp\left(\frac{-i}{a\cdot G}\right),R_{2}<ST\\ x_{i,j}^{t}+Q\cdot L,R_{2}\geq ST \end{array}\right.$$     End     For i = (PN + 1): N         Adjust each sparrow’s position by Equation (17).
(17)$$\left.X_{i,j}^{t+1}=\left\{\begin{array}{l} Q\cdot exp\left(\frac{X_{worst}^{t}-X_{i,j}^{t}}{i^{2}}\right)\;i>\frac{N}{2}\\ X_{p}^{t+1}+\left|X_{i,j}^{t}-X_{p}^{t+1}\right|\cdot A^{+}\cdot L\;otherwise \end{array}\right.\right.$$     End for     For i = 1: SN         Adjust each sparrow’s position by Equation (18).
(18)$$X_{i,j}^{t+1}=\left\{\begin{array}{cc} X_{best}^{t}+\beta \cdot \left|X_{i,j}^{t}-X_{best}^{t}\right|, & {\;\;\;\;f}_{i}>f_{g}\\ X_{i,j}^{t}+k\cdot \left(\frac{\left|X_{i,j}^{t}-X_{worst}^{t}\right|}{\left(f_{i}-f_{w}\right)+\varepsilon }\right), & {\;\;\;\;f}_{i}=f_{g} \end{array}\right.$$     End     Get the current new location;     If the new location is better than before, update it;     t = t + 1 End while 3: Return K and α

**Figure 2:**
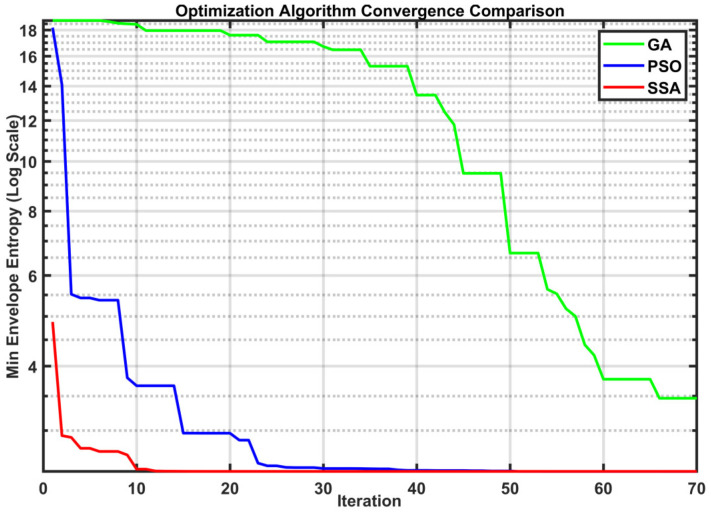
Convergence comparison of GA, PSO, and SSA.

The SSA-optimized VMD can adaptively determine the optimal configuration of *K* and α. Using envelope entropy as a fitness function allows the algorithm to handle non-stationary signals better. The SSA has a simple structure and few parameters. It requires less computing power for optimization. Therefore, it completes the process quickly. As illustrated in Figure 2, the convergence behaviors of genetic algorithm (GA), particle swarm optimization (PSO), and SSA were compared under identical experimental conditions.

GA (green line) exhibits the slowest convergence rate, with a significant lag period during the first 40 iterations, indicating low search efficiency in the early stages. PSO (blue line) demonstrates a faster initial descent but exhibits a characteristic “staircase” convergence pattern. As shown in the intervals of iterations 10–15 and 22–30, the algorithm frequently stagnates in local optima (premature convergence) and struggles to escape, re-quiring significantly more iterations to match the accuracy of SSA.

In contrast, the SSA (red line) demonstrates the most superior performance. It achieves a sharp, near-vertical descent in the first 5 iterations, reaching the global optimum without stagnation. This indicates that SSA possesses extremely strong global optimization capabilities and high convergence speed.

### 3.3. IMF Screening on the Basis of SE

This work uses SE to screen the IMFs obtained after VMD to separate the noise signals. SE serves as a quantitative metric for assessing signal complexity and predictability, with the advantage of being less parameter-dependent while providing high estimation accuracy. It is particularly well suited for handling nonstationary signals. Noise primarily affects signals by disrupting their regularity and increasing their complexity. Consequently, the noise content in a signal is positively correlated with its SE. A lower SE indicates stronger signal regularity, whereas a higher SE reflects greater structural complexity and stronger randomness in the sequence. For a given signal $X=\left[x_{1},x_{2},{\cdots ,x}_{N}\right]$ of length N, the SE calculation process is as follows.

Step 1: Construct an *m*-dimensional vector $X_{i}=\left[x_{i},x_{i+1},\cdots ,x_{i+m-1}\right]$ from the original signal and calculate the Chebyshev distance between two vectors $X_{i}$ and $X_{j}$.

(19) $$d\left[X_{i},X_{j}\right]=max\left(\left[\left|x_{i+k}-x_{j+k}\right|\right]\right).$$

Here, k=0,1,2⋯,m−1,i,j=0,1,2,⋯,N−m+1,i≠j. *m* is usually taken as 2.

Step 2: Calculate the matching counts. Set the similarity tolerance r. A small r (near 0) leads to almost no vector matches. The results become unstable. They are also sensitive to noise. In contrast, a large r causes almost all vectors to match. This process loses the detailed information of the sequence. Usually, it is taken as 0.1 to 0.25 times the standard deviation of the original sequence. For a given vector $X_{i}$, the number of vectors $X_{j}$ whose distance to $X_{i}$ is less than or equal to r is denoted as $B_{i}^{m}$.

Step 3: Calculate the probability estimation. In the m-dimensional space, compute the average of $B_{i}^{m}$ for all *i*, denoting the probability estimate of similarity between any two vectors as $B^{m}(r)$.

(20) $$B^{m}(r)=\frac{\sum_{i}B_{i}^{m}(r)}{\left(N-m+1\right)}/\left(M-m+1-1\right)=\frac{\sum_{i}B_{i}^{m}(r)}{\left(N-m+1\right)}/\left(M-m\right).$$

Here, N−m+1 represents the number of $d\left[X_{i},X_{j}\right]$. Similarly, we can calculate the probability estimate for the m−1 dimension by Equation (21). The calculation formula for SE is shown in Equation (22). This paper implements an automatic threshold based on the statistical distribution of SE, with the threshold T calculated as Equation (23)

(21) $$A^{m+1}\left(r\right)=\frac{\sum_{i}A_{i}^{m+1}(r)}{\left(N-m\right)}/\left(M-m-1\right).$$

(22) $$SE=-ln\left[A^{m+1}(r)/B^{m}(r)\right].$$

(23) $$T={SE}_{mean}+\beta \cdot {SE}_{sd}.$$

Here, ${SE}_{mean}$ and ${SE}_{sd}$ represent the mean and standard deviation of all SEs, and $\beta \in \left[0.5,2\right]$. A smaller β leads to more aggressive denoising. In contrast, a larger β results in more conservative denoising. β is usually set to 1.

The noise-dominant IMFs increase the overall mean of the SE. Consequently, IMF components exhibiting values significantly higher than this average are identified as noise. SE screening cooperates with the PSE screening method described in Section 3.2, forming a comprehensive IMF screening framework. The PSE screening mechanism performs preliminary classification of IMFs from a frequency domain perspective, whereas SE screening conducts secondary filtration on the basis of signal complexity and predictability. This complementary approach enables a more thorough and accurate classification of IMF components. Such an integrated methodology is crucial for enhancing the quality and stability of MEMS gyroscope signals, thereby better satisfying precision requirements in practical applications. Finally, the algorithm reconstructs the signal by combining useful signals obtained from EMD and VMD.

## 4. Experiments and Results

In this study, the validation is performed using a vibratory MEMS gyroscope based on the Coriolis force. However, the proposed algorithm is data-driven. It relies on the statistical characteristics of the signal (such as entropy and mode decomposition) rather than the specific mechanical structure of the sensor. Therefore, the proposed method is theoretically applicable to other gyroscope architectures that exhibit similar noise patterns.

### 4.1. Experimental Processing of Simulation Signals

**Figure 3:**
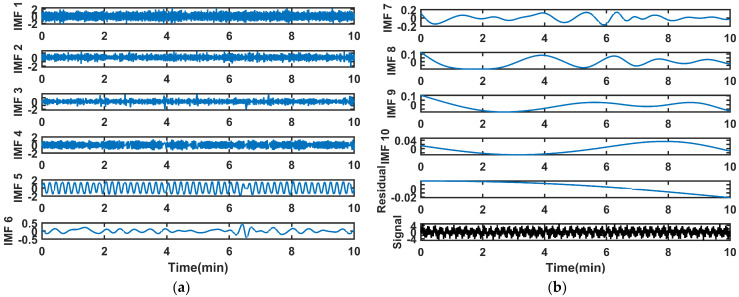
The EMD results of the signal: (**a**) IMF1~IMF6; (**b**) IMF7~IMF10, residual, and original signal.

We generate a pure simulated gyroscope signal. Then, we add Gaussian noise, quantization noise, and colored noise to create the test data. We conducted algorithm tests on the simulated data in the MATLAB environment at a sampling rate of 1000 Hz for 10 min. The original signal undergoes first-layer decomposition via EMD. The results are in Figure 3. The high-frequency IMFs exhibit more pronounced fluctuations and may contain more noise components. The low-frequency IMFs exhibit relatively gentle changes and are more likely to contain useful information from the original signal.

**Figure 4:**
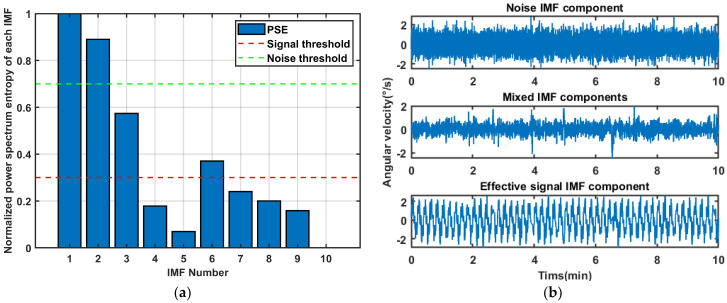
The classification results of IMFs after EMD of simulated signal. (**a**) Normalized power spectrum entropy of each IMF; (**b**) IMF classification results.

We calculate the PSE of each IMF for classification. The results are in Figure 4. EMD-IMF1~IMF2 exhibit high PSE and vibration frequency, indicating that they are noise IMFs. EMD-IMF3 and EMD-IMF6 are identified as mixed signals containing both noise and useful information. EMD-IMF4~IMF5 and EMD-IMF7~IMF10 are pure and their PSE are regular, classifying them as useful IMFs.

**Figure 5:**
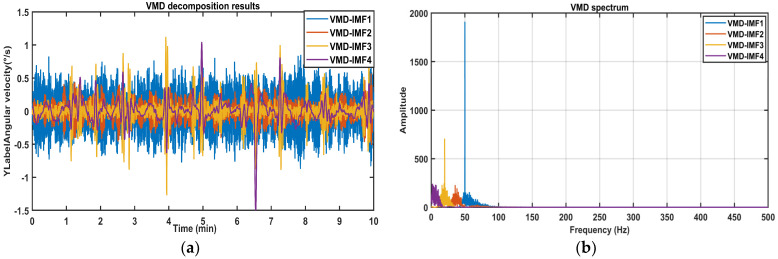
The VMD results of the signals: (**a**) IMF time domain diagram; (**b**) IMF spectrum diagram.

Following Section 3.2, the mixed signals are subjected to a secondary decomposition, with the results presented in Figure 5. Signals are further decomposed into four new IMFs. Among these, VMD-IMF1 exhibits disordered waveforms, significant amplitude variations, and higher frequencies, indicating that they are likely noise. In contrast, VMD-IMF2~IMF4 are concentrated in the low-frequency band with stable waveforms, showing strong agreement with the trend variations of the original signal.

**Figure 6:**
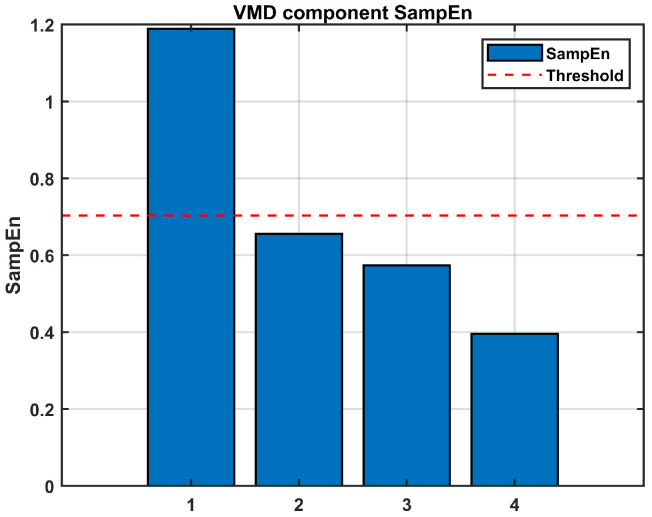
The sample entropy of IMFs after VMD of the mixed signals (Simulated signal).

Following Section 3.3, the SEs of the four new IMFs are calculated for classification, with the results shown in Figure 6. The SEs of VMD-IMF1 surpasses the established threshold, confirming that they are noise. Conversely, the SEs of VMD-IMF2~IMF4 are below the threshold, confirming that they are valid signals. This secondary decomposition effectively achieves the separation of noise from useful signals within the mixed component, thereby further enhancing the accuracy of signal reconstruction. After the classification of IMFs and separation of noise components, the gyroscope signal is reconstructed.

**Figure 7:**
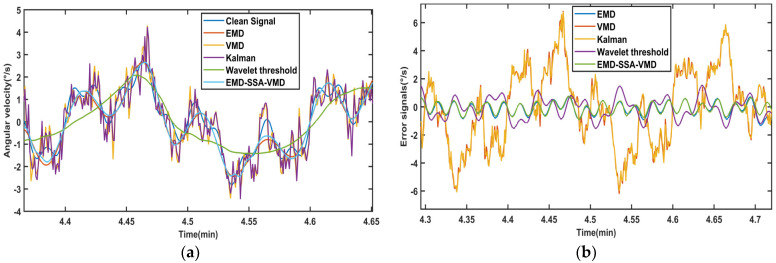
The denoising results of different methods for signals: (**a**) The local comparison of the denoising signals. (**b**) The local comparison of error signals.

To validate the proposed method, we compare it with EMD-Wavelet, SSA-VMD, KALMAN, and WTD, as shown in Figure 7. The signal waveform processed by our algorithm more closely aligns with the original signal. The error signals graph shows that the reconstruction error of the algorithm in this paper is also minimal.

To quantitatively evaluate the performance of the five algorithms, we conducted a quantitative analysis using the SNR and RMSE, as shown in Table 1. Parameter settings and optimization strategies for all methods are in Table 2. The results show that our algorithm is more advantageous, indicating that the algorithm in this article can better suppress noise in the signal. Meanwhile, it can also preserve useful information in the original signal as much as possible. We analyze the computational efficiency. The EMD-Wavelet, KALMAN, and WTD are fast. However, the proposed method takes a relatively long time. This is due to the multiple iterations required by the SSA to find optimal parameters. Although it consumes more time, it achieves the highest accuracy. This trade-off is acceptable for offline engineering applications where precision is the priority.

**Figure 8:**
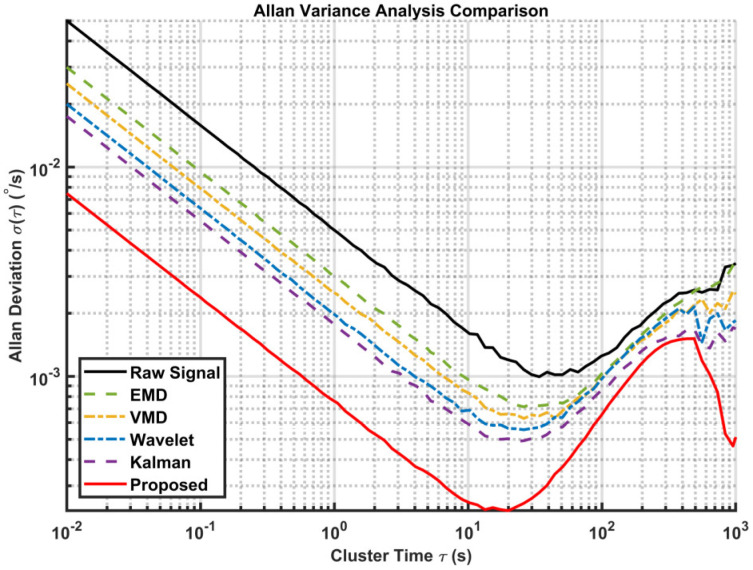
Comparison of Allan variances of different algorithms (Simulated signal).

Standard noise analysis methods, such as the Allan variance, are widely used to decouple different error sources [38,39]. We use the Allan variance to evaluate the effectiveness of the algorithm in suppressing random errors, as shown in Figure 8. The curve of the proposed method is consistently located at the bottom, indicating the lowest overall noise level across all time clusters. In the short cluster time region, the slope of the proposed method aligns with −0.5 but with a significantly lower magnitude compared to other methods. This demonstrates the superior capability of the proposed dual-layer strategy in suppressing high-frequency quantization noise. Specifically, the bias instability is reduced significantly, proving that our method effectively minimizes random drift without distorting the underlying signal trend. The specific quantitative parameters extracted from this plot are listed in Table 3.

### 4.2. Experimental Processing of Real Gyroscopic Signals

**Figure 9:**
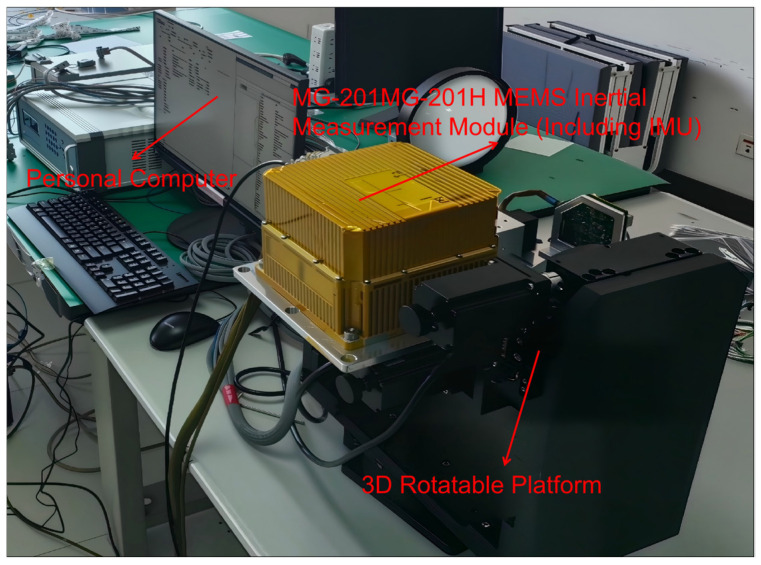
Experimental device for gyroscope data acquisition.

To validate the practical performance of our algorithm, we gathered actual gyroscope signal data to conduct experiments. The experimental device used for gyroscope data acquisition is shown in Figure 9. The experiment utilized an MG-201H MEMS inertial measurement (Manufacturer name: Shaanxi Xinrong Jiaye Technology Co., Ltd.; Mei County, Baoji City, Shaanxi Province, China) unit mounted on a three-axis rotation platform for data acquisition. Our research object is the angular velocity measured by IMU during platform motion. After initializing the gyroscope, we set the data output rate to 1000 Hz and collected 1 h of gyroscope data as a sample.

**Figure 10:**
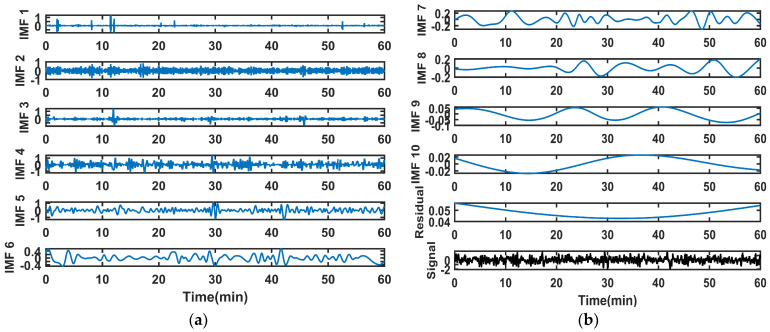
The EMD results of the gyroscope signal: (**a**) IMF1~IMF6; (**b**) IMF7~IMF10, residual.

The angular velocity variation trajectory of the platform motion is a sine wave. The same experiment was conducted as in Section 4.1. The EMD results of the gyroscopic signal are shown in Figure 10.

**Figure 11:**
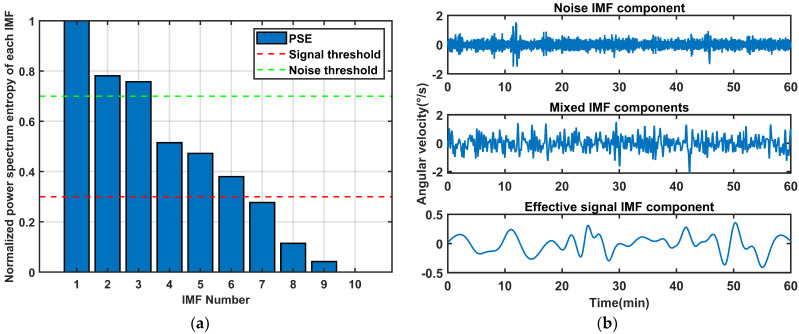
The classification results of IMF after EMD of gyroscope signals: (**a**) Normalized power spectrum entropy of each IMF. (**b**) IMF classification results.

Similarly, the IMFs were separated according to the screening mechanism in Section 3.2, as shown in Figure 11. IMF1~IMF3 were identified as noise signals, IMF4~IMF6 were classified as mixed signals, and IMF7 to IMF10 were determined to be useful signals.

**Figure 12:**
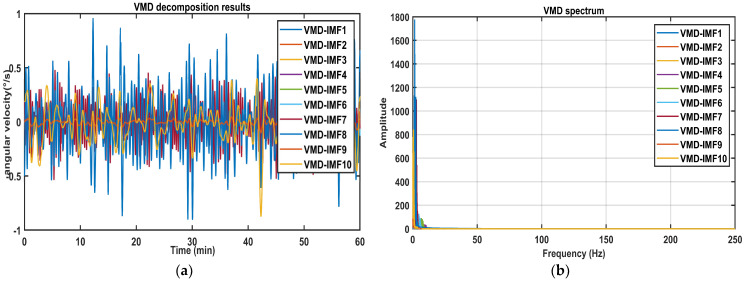
The VMD results of the mixed signals: (**a**) Time domain diagram. (**b**) Spectrum diagram.

Then, the mixed signals were further decomposed via VMD, and the results are displayed in Figure 12. The mixed signals undergo decomposition, yielding several new IMFs, which are then used for further signal analysis.

**Figure 13:**
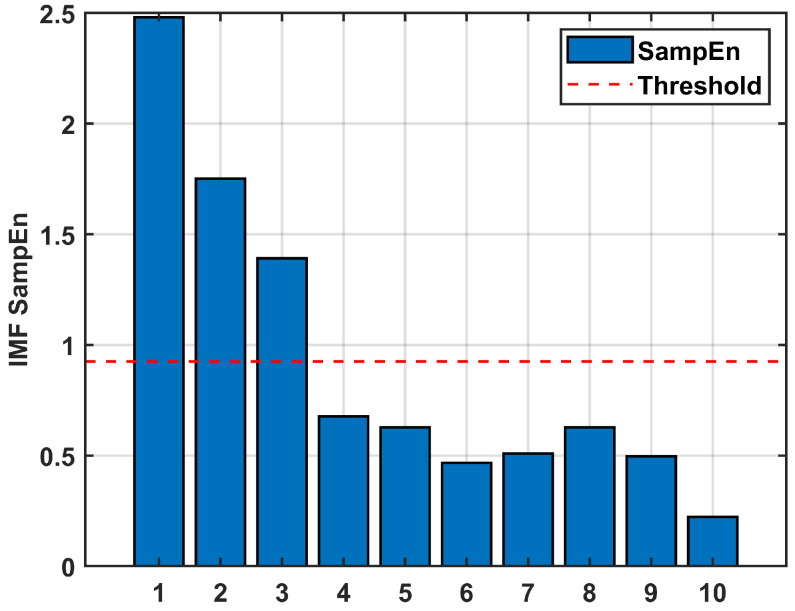
The sample entropy of IMFs after VMD of the mixed signals (Gyroscope signal).

The IMFs obtained from VMD are classified as shown in Figure 13. VMD-IMF1~IMF3 are classified as noise, whereas VMD-IMF4~IMF10 are classified as useful signals. After completing the IMF classification and noise separation, the signal is reconstructed.

**Figure 14:**
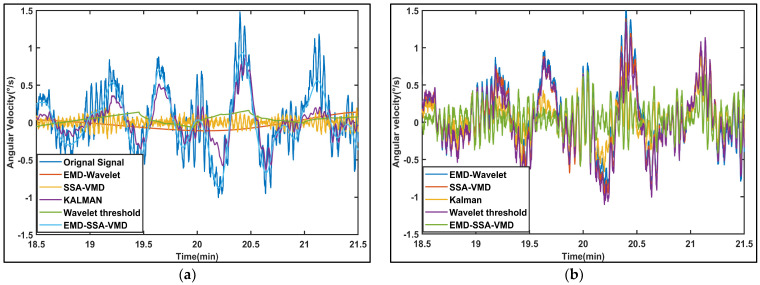
Comparison of five denoising methods on the gyroscope signal: (**a**) The local comparison of the denoising signals. (**b**) The local comparison of residual signals.

The comparison of the denoising performance across the five algorithms is provided in Figure 14. The signal processed by our method closely matches the original signal in terms of waveform, with fluctuation patterns that more closely approximate the true characteristics of the original signal. It indicates superior preservation of the primary information contained in the original signal, thereby minimizing reconstruction errors. In contrast, the other methods suffer from excessive smoothing processing, resulting in significant deviations from the original waveform. This indicates that there is too much information loss in the original signal, resulting in significant reconstruction errors. The residual signal graph indicates that our algorithm does not have excessive smoothing.

Since the true angular velocity is unknown for the real experiments, the standard time domain SNR cannot be calculated directly. To rigorously evaluate the noise suppression performance, we adopt a Spectral Power Integration method based on the physical bandwidth of the gyroscope signal. The proposed method is based on two fundamental premises. First, the effective motion signals of MEMS gyroscopes are typically concentrated in the low-frequency band. Second, sensor noise is generally distributed across the entire frequency spectrum or predominantly in the high-frequency range. Therefore, this approach relies on the inherent physical characteristics of the signal rather than the output of the denoising algorithm. We define the effective signal bandwidth using a cutoff frequency, $f_{c}$. It is based on the spectral energy distribution of the original signal (where 99% of the energy is concentrated below $f_{c}$). The signal power $P_{signal}$ and noise power $P_{noise}$ are estimated by integrating the power spectral density (PSD) over separate frequency bands. The SNR is calculated as follows:

(24) $${SNR}_{est}=10log10\left(P_{signal}/P_{noise}\right)$$

(25) $$P_{signal}=\int_{0}^{f_{c}}PSD\left(f\right)df$$

(26) $$P_{noise}=\int_{f_{c}}^{f_{Nyq}}PSD\left(f\right)df$$

Here, PSD(f) is the PSD of the signal, $f_{c}$ is the cutoff frequency, and $f_{Nyq}$ is the Nyquist Frequency. This metric strictly separates the low-frequency motion components from the high-frequency sensor noise in the frequency domain, avoiding the assumption that the removed components are pure noise. The calculation results are in Table 4.

**Figure 15:**
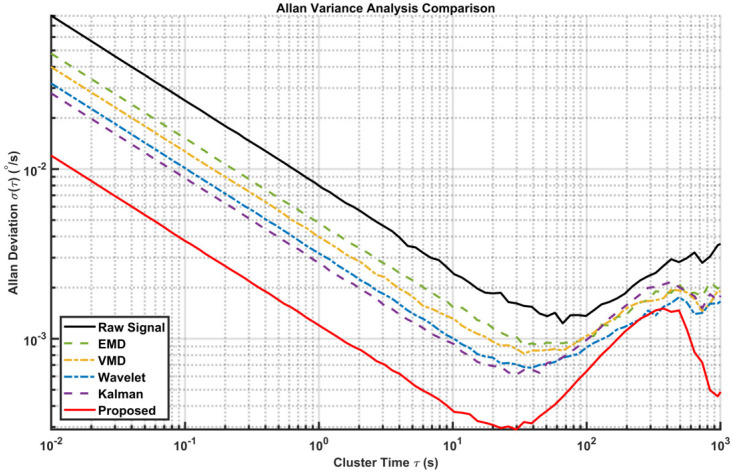
Comparison of Allan variances of different algorithms (Gyroscope measurement signal).

We also calculated the Allan variance of the actual signal, as shown in Figure 15. The specific quantitative parameters extracted from this plot are listed in Table 5. The analysis confirms that the proposed method not only suppresses high-frequency noise but also improves mid-term bias stability.

The results of simulation experiments and real signal experiments show that the proposed EMD-SSA-VMD denoising algorithm achieves excellent performance in terms of both noise reduction effectiveness and signal reconstruction accuracy.

## 5. Conclusions

This paper presents a denoising method based on EMD-SSA-VMD to remove random noise from MEMS gyroscope signals and improve accuracy. We compared this method with traditional EMD, VMD, and WTD. It performs better in terms of SNR and RMSE. The algorithm relies on a dual-layer decomposition structure using EMD and VMD. It also employs a dual-level screening process with PSE and SE, along with SSA for optimization. This approach combines the speed of EMD with the ability of VMD to stop modal aliasing. We use SSA to achieve adaptive optimization of modal decomposition parameters. The dual-level screening effectively separates the noise. The results of the experiment confirm that our algorithm is effective and superior for denoising gyroscope signals. It separates noise from useful information accurately. It also preserves the features of the original signal during reconstruction. The algorithm significantly improves the spectral SNR and reduces the Noise Floor in dynamic conditions, which directly translates to better signal quality for motion tracking applications. This offers a valuable reference for high-precision offline measurements in practical engineering.

## Figures and Tables

**Table 1:** The test results of five experimental schemes for simulating signals.

Method	SNR (dB)	RMSE	Runtime (s)
Original Signal	17.24	0.6486	-
EMD-Wavelet	27.92	0.3508	0.9875
SSA-VMD	24.21	0.4343	75.6395
KALMAN	34.81	0.2359	0.485
Wavelet Threshold	18.83	0.5917	0.738
**EMD-SSA-VMD**	**48.16**	**0.1** **094**	**49.15**

**Table 2:** Parameter settings and optimization strategies for all methods.

Method	Key Parameter	Value	Selection Justification
EMD-Wavelet	IMF Selection Denoising Strategy	IMF 1∼IMF 3 Soft threshold	Based on energy spectral density Only the noise-dominant IMFs
SSA-VMD	Population/Iteration Search Range	30/20 K ∈ [3, 12], α ∈ [100, 3000]	Search capability, computational cost Finds the global optimum for the signal
KALMAN	State Model (A, H) Covariance (Q, R)	$A=I_{2\times 2}$, $H=\left[1,\;1\right]$ R $\approx \sigma_{static}^{2}$ *Q*/*R* $=10^{-2}$	Standard random walk model R is fixed by static noise variance; Q is manually tuned to balance
Wavelet Threshold	Wavelet Basis Decomposition Level	sym8 5	High similarity to gyro signal features determined via trial-and-error

**Table 3:** Allen variance quantitative error analysis results.

Algorithm	ARW (deg/sqrt(h))	Bias Instability (deg/h)	RRW deg/h/sqrt(h)
Signal	0.2915	5.3965	0.7130
EMD	0.1761	3.8733	0.7194
SSA-VMD	0.1471	3.4097	0.5880
Wavelet Threshold	0.1154	3.0119	0.7491
KALMAN	0.1026	2.6538	0.6375
**EMD-SSA-VMD**	**0.0446**	**1.2376**	**0.3950**

**Table 4:** The test results of four experimental schemes for gyroscope signals.

Method	SNR(dB)
Original Signal	5.4653
EMD-WTD	8.7513
SSA-VMD	7.0717
KALMAN	7.2487
Wavelet threshold denoising	9.1971
**EMD-SSA-VMD**	**15.5771**

**Table 5:** Allan variance quantitative error analysis results.

Algorithm	ARW (deg/sqrt(h))	Bias Instability (deg/h)	RRW deg/h/sqrt(h)
Signal	0.4633	6.6808	0.8535
EMD	0.2806	4.9487	0.6462
SSA-VMD	0.2325	4.4253	0.6498
Wavelet Threshold	0.1860	3.6525	0.5542
KALMAN	0.1638	3.3164	0.6131
**EMD-SSA-VMD**	**0.0699**	**1.5710**	**0.3993**

## Data Availability

The data presented in this study are available on request from the corresponding author. Due to confidentiality agreements in our lab, we are unable to share the raw data at this time.

## Abbreviations

The following abbreviations are used in this manuscript:

MEMS	Microelectromechanical systems
EMD	Empirical mode decomposition
VMD	Variational mode decomposition
SSA	Sparrow search algorithm
IMFs	Intrinsic mode functions
PSE	Power spectral entropy
SE	Sample entropy
WTD	Wavelet threshold denoising
EE	Envelope entropy
SNR	Signal-to-noise ratio
RMSE	Root mean square error
PSD	Power spectral density
